# The Impact of Energy and Protein Levels on Yellow Mealworm Growth and Chemical Composition

**DOI:** 10.3390/insects17020221

**Published:** 2026-02-20

**Authors:** Marko Vukadinović, Mirko Ivković, Dejan Beuković, Miloš Petrović, Miroslava Polovinski Horvatović, Nikola Laćarac, Saša Krstović, Igor Jajić

**Affiliations:** 1 Department of Animal Science, Faculty of Agriculture, University of Novi Sad, Trg Dositeja Obradovića br. 8, 21000 Novi Sad, Serbia; marko.vukadinovic@stocarstvo.edu.rs (M.V.); dejan.beukovic@stcarstvo.edu.rs (D.B.); miroslava.polovinski@stocarstvo.edu.rs (M.P.H.); sasa.krstovic@stocarstvo.edu.rs (S.K.); igor.jajic@stocarstvo.edu.rs (I.J.); 2 Department of Entomology and Plant Protection, Faculty of Agriculture, University of Novi Sad, Trg Dositeja Obradovića br. 8, 21000 Novi Sad, Serbia; milos.petrovic@polj.edu.rs (M.P.); nikola.lacarac@polj.edu.rs (N.L.)

**Keywords:** *Tenebrio molitor*, protein, energy, diets, chemical composition

## Abstract

To maximize protein production from insects, it is essential to understand their nutritional requirements, production characteristics, and the chemical properties of the resulting feed, along with how these factors depend on the insects’ diets. This research involved the use of nine different insect diets, all based on wheat bran. Corn and soybean meal were included to enhance the energy and protein content. The weight gain of larvae was significantly affected by the level of protein in their feed; however, the energy level did not have a significant impact on the weight gain measured in grams per day per tray. This gain ranged from 0.57 g/day per tray for insects fed low-energy, low-protein diets to 0.71 g/day per tray for those fed high-protein, medium-energy diets. Feed conversion ranged from 1.83 to 2.34 and was significantly influenced by energy and protein levels. The chemical composition of reared insects showed significant differences, particularly in fat content. The fat content ranged from 38.5% with a low-protein diet to just 26.9% with a high-protein diet. In contrast to fat content, the variability in protein content was smaller, ranging from 44.8% in a low-protein, high-energy diet to 51.9% in a high-protein, high-energy diet.

## Simple Summary

In this study, we examined how different diets affect yellow mealworm larvae production. Nine diets with varying energy and protein levels were tested. Larval weight gain depended mainly on protein content, while energy level had no significant effect. Feed conversion efficiency was influenced by both energy and protein levels. Diets strongly affected insect composition, especially fat content, which decreased as dietary protein levels were increased. Protein content varied less than fat content, and was highest in insects fed high-protein, high-energy diets.

## 1. Introduction

Numerous insect species have undergone a remarkable transformation in their societal roles. Most recognizable are species belonging to the order Coleoptera and family Tenebrionidae: *Tenebrio molitor* (yellow mealworm) and *Alphitobius diaperinus* (lesser mealworm [1]. Once regarded solely as stored product pests, causing significant damage to grains and other commodities, these insects have now earned recognition as sustainable and nutritious edible candidates [2]. Similar to beetles, species from the order Orthoptera, such as *Acheta domesticus*, common household pests [3], and *Locusta migratoria*, dangerous polyphagous pests [4], were traditionally considered harmful. However, over the last two decades, they have undergone a role transition and are now viewed as ideal candidates for mini-livestock [5]. Numerous studies highlight the urgent need for protein diversification and the expansion of protein sources worldwide. Protein-rich food and feed are often criticized as unsustainable due to their environmental impact, including extensive use of arable land and significant greenhouse gas emissions [6]. This has pushed lawmakers and policymakers to develop greener strategies. Insects have emerged as a promising solution, filling the gap between the increasing global demand for protein expected to rise sharply as the population exceeds 9 billion in the next 25 years and the inability of conventional protein sources to meet both consumer needs and environmental sustainability goals [7,8,9].

The idea that insects are a new protein source became a controversy two decades ago in numerous Western countries, contrary to Asia, Central and Southern American countries, where over 2 billion people consume insects for ages, either regularly or as a part of traditional meals.

Since 2010, the idea of consuming insects either as food or feed has become more appealing to users in the named traditional societies [10,11]. The first introduction of people to edible insects occurred when they started buying insects as food for reptiles, birds, and pet fish. The next wave of insect usage was as a proper alternative protein in feed mixtures used for fish, poultry and pig farming, where the people as consumers were referred to as secondary consumers of insects [12]. The final and up-to-date wave began when the European Food Safety Authority marked at *T. molitor* [13] first and then followed *L. migratoria* [14], *A. domesticus* [15] and *A. diaperinus* [16] as species safe for human consumption. The insect properties, such as their adaptability, rapid reproduction rates, and nutritional profiles rich in protein, healthy fats, vitamins, and minerals, made them ideal for contributing to food security while addressing environmental challenges, which became a hurdle for traditional protein sources [16,17]. This shift from being pests toward being intentionally cultured and farmed underscores their potential for insects to redefine themselves, not as nuisances, but as valuable resources in the quest for sustainable animal and human consumption. In addition to these four species, other insect species belonging to the order Diptera, *Hermetia illucens* and *Musca domestica*, have also been recognized, but solely for use in animal feed [18,19,20].

However, among all the mentioned species, *T. molitor* has stood out as an excellent candidate for large-scale farming [21]. This species is considered low-maintenance compared to the *Diptera* and *Orthoptera* species. The rearing of the larvae is fully aligned with the vertical farming strategy. The environmental requirements are much more rudimentary compared to other species as the yellow mealworm requires suitable temperature and humidity, while the suitable light requirements are completely omitted because of this species’ biology [22]. As a secondary stored product pest, this species has proven adaptable for farming on various agricultural products and sidestreams, provided that the substrate’s humidity is appropriate. One drawback that distinguishes yellow mealworm from other edible insect species is its relatively long life cycle, with a full generation lasting from 70 to 110 days [22,23,24].

The chemical composition of the feed used for the rearing is often crucial for the length of the development and the yield of the larvae. In the field of animal nutrition, the energy and protein requirements of domestic animals are well understood. Properly balancing these two factors can lead to lower production costs and a more favorable chemical composition of the final product. In this experiment with *T. molitor*, we aimed to achieve a similar goal: to reduce costs while ensuring an optimal chemical composition. Larvae growth and performance are strongly influenced by diet, making the formulation of an efficient diet crucial for insect semi and mass production. Research by Adámková et al. [22] has shown that dietary adjustments can improve larval protein and fat content, reinforcing the importance of precise substrate formulation for optimal production outcomes. The protein and energy levels in the rearing substrate play a key role in optimizing *T. molitor* larvae production and reducing costs. A well-balanced intake of these nutrients directly affects growth, development, and chemical composition, enhancing their value as an alternative protein source for animal and human nutrition.

The most common feeding substrates used in research and mass production are locally available byproducts, often lacking standardized nutritional profiles. This variability affects the biological cycle duration and the nutritional composition of insect-derived products. Yellow mealworm, an omnivorous insect, can break down cellulose, a major component of fiber-rich waste. The abundance of these cellulose-rich byproducts is closely tied to local production sources. While cellulose is not a primary growth factor, the protein and energy content, along with their ratio in the substrate, are crucial for successful *T. molitor* rearing.

The nutritional requirements of *T. molitor* are not well understood [25]. The protein and energy ratio in feeding substrates is crucial for efficiently rearing *T. molitor* because it directly influences their growth rate, survival, and nutritional composition. Striking the right balance between protein and energy is key to optimizing larval growth and nutritional composition. A balanced protein-to-energy ratio ensures that the larvae receive adequate nutrients for optimal development, as protein is essential for tissue building and energy supports metabolic activities. An imbalance, such as excessive energy with insufficient protein, can lead to suboptimal growth and reduced protein content in the larvae, which diminishes their value as a sustainable protein source [26]. On the contrary, when the diet is rich in protein but lacks sufficient energy, the larvae may experience slower metabolic processes, as their energy demands are not adequately met to sustain active growth and physiological functions. While the high protein content supports tissue development and contributes to their nutritional value, the energy deficiency can reduce overall efficiency in feed conversion ratio (FCR), leading to lower biomass production. This imbalance can also increase stress on the larvae, potentially affecting survival rates and the overall productivity of rearing. Therefore, carefully optimizing this ratio in substrates is key to achieving high-quality yields in mealworm farming.

A study conducted by Jajić et al. [27] found that *T. molitor* larvae contain a high level of crude protein (55.83% dry matter content), making them a potentially valuable protein source in animal nutrition. Feeding larvae with protein-rich substrates can increase their protein content, but only up to some point, thereby improving their nutritional value. Research has shown that larvae raised on high-protein substrates achieve higher yields and a superior amino acid profile, making them particularly suitable for monogastric animal feed [28]. Energy within the substrate, primarily provided by carbohydrates and fats, is essential for larval metabolic processes. Adequate energy intake allows larvae to efficiently utilize proteins for growth, whereas an energy deficit may force them to use proteins as an energy source, reducing their availability for tissue development. Therefore, maintaining a proper balance between protein and energy is vital for optimizing production.

This study was conducted to gain a deeper understanding of the protein and energy requirements of mealworm larvae for optimal growth and cost-effective production. Three levels of protein and three energy levels were combined in the feed mixtures used as a substrate for rearing the larvae. Wheat bran was used as the base for each feed mixture, as it best meets the nutritional needs of the yellow mealworm. Our trial aimed to optimize production so that the results could be applied under large-scale production conditions, which is why the production parameters per container were closely monitored weekly. This study aimed to evaluate the effects of dietary protein and energy levels on the production parameters and body composition of larvae of the *Tenebrio molitor*. The emphasis was on growth rate as the effect of the dietary protein level, body fat deposition in relation to dietary energy level, and their interaction, to identify nutritionally and economically optimal nutritional combinations.

## 2. Materials and Methods

### 2.1. Feed

Nine different feeds were used, formulated with three different levels of protein and three different levels of energy. Wheat bran, commonly used as a control feed in experiments with the yellow mealworm, served as the base for each mixture, while pure wheat bran was used as the control treatment. To increase the protein levels compared to the control, soybean meal was added, as it is one of the most commonly used protein sources in animal feed. Corn, widely used as an energy source in animal nutrition, was included to raise the energy level. Contrary to corn, sugar beet pulp was used to dilute the energy levels. The compositions of the raw materials is presented in Table 1, while their proportions in the experimental diets are given in Table 2. In the purpose of assessing the energy levels of mixed meals, an estimate of metabolic energy from Pascual et al. [29] was used, where the digestibility energy (MJ/kg DM) for *Tenebrio* was determined for corn, soybean meal, and wheat bran. The nutrient content of each complete diet was calculated as the weighted sum of the contributions of its individual ingredients, based on their inclusion levels, which is a standard practice in the formulation of animal diets. By varying the proportions of these feed components, treatments were formulated to provide three different protein levels, each combined with three different energy levels. To supply water agar gel consists from 99% of water 1% dry matter, which was based on agarose and agaropectin, was used per tray. Agar gel was prepared by dissolving 10 g of agar (AGAR, Torlak, Serbia, purity p.a.) in 1 L of distilled water, and then sterilized in an autoclave SUTJESKA TIP 300 (Sutjeska, Belgrade, Serbia). The results of the chemical analyses of the feed mixtures used in the experiment are presented in Table 3.

### 2.2. Insects

At the start of preparation for the trial, ten-day-old adults were put on substrate in rearing trays, dimensions 50 × 60 × 10 cm, and they were left for 48 h to copulate and lay eggs. After those 48 h, the adults were removed through sieving, and the rearing trays were put in a chamber with controlled temperature and humidity, with the mentioned parameters at 26 ± 1 °C and 65 ± 5% Rh. Seven days after adult removal, freshly cut carrots and potatoes were provided to the newly emerged larvae and replaced every two days. Rearing lasted 42 days, as the starting day was counted when neonate larvae were detected in the substrate. At the end of pre-experimental rearing, they were sieved through the systems of vertical sieves whose cells ranged from 0.75 to 1.25 mm to separate them from frass and substrate that was not consumed. Pure larvae assay was transferred to a clean rearing tray and left for 24 h to fast and excrete the remaining ingested substrate.

The insects were standardized before the start of the trial. All larvae were hatched within a three-day time span. To determine the average larval weight at the beginning of the trial, three measurements were taken, each consisting of exactly 50 larvae, and the mean weight and coefficient of variation were calculated.

### 2.3. Experimental Design

The aim of the trial was to determine the production performance of yellow mealworm larvae, with the large-scale production of the larvae in mind. The trial lasted five weeks, until the appearance of the first pupae, and it ended at the same time for all treatments. Each of nine treatments had 4 replicates, and each replicate contained 2 g of yellow mealworm larvae (approximately 252 larvae per tray), stored in a plastic tray measuring 11 × 6.5 × 5.5 cm, with a surface area of 60.5 cm^2^. Each replicate began with 3 g of feed. Throughout the trial, the same amount of feed was provided, with a total of 44 g of feed being provided to larvae per tray until the end of the trial. The same amount of agar was added to each replicate every other day. All larvae were kept under identical environmental conditions (26 ± 1 °C, 65 ± 5% RH), most of the time in total darkness, except during sieving, feeding, or agar supplementation. Standard production parameters were monitored weekly, including mass per tray (i.e., per replicate), weight gain, and feed conversion. The trial was repeated twice. At the end of the first trial, the larvae were analyzed for chemical composition.

Feed Conversion Ratio (FCR, g/g) = Diet intake, g / (larval biomass at the end, g-larval biomass at the start, g)

Protein Conversion Ratio (PCR, g/g) = (Diet intake, g × Diet protein, g/g) / (Larval weight gain, g × Larval protein, g/g)

Parameters such as weight gain and feed intake are expressed as grams per day per tray, similar to those in the manuscript by Pascual et al. [29]. All measurements of larvae conducted throughout the duration of the experiment were based on wet body mass, and the obtained data were subjected to statistical analysis. Prices of wheat bran, soybean meal, corn grain, and sugar beet pulp, used for the economic analyses, were 0.22, 0.51, 0.19, and 0.28, respectively, according to the local Serbian market in May 2025.

### 2.4. Chemical Analysis

The content of dry matter was determined after drying (AOAC Official Method 934.01) [30]. Crude protein was analyzed using the standard Kjeldahl method (AOAC Official Method 2001.11) [31] with a conversion factor of 6.25. Crude fat content was determined as petroleum ether extract (AOAC, Official Method 991.36) [32], while ash content was determined in the furnace at 600 °C (AOAC Official Method 942.05) [33]. Neutral detergent fiber (NDF) and Acid detergent fiber (ADF) were determined on the ANKOM DELTA fiber analyzer (ANKOM Technology, Macedon, NY, USA) by applying methods provided by the manufacturer (ADF Method, 2017; Ankom Technology 2017) [34]. Hemicelluloses were calculated by subtracting ADF from NDF. All chemical analysis were performed in duplicate.

### 2.5. Statistical Method Description

For statistical analysis of collected data on weight gain, feed intake, and FCR, a factorial Analysis of Variance (ANOVA) was performed using Generalized Linear Model (GLM), with protein and energy levels as fixed factors and trial as a random factor. In the case of the chemical composition of larvae and protein conversion ratio (PCR), a simpler model was used. Again, it was a factorial ANOVA performed using the GLM, with two fixed factors, protein and energy, and three levels of each. This model excludes the random effect of trial since the chemical composition of larvae was determined just once, in the first trial, and the same data was necessary for the calculation of PCR. Results are presented as means with model-based pooled standard error of the mean (SEM). Normality and equality of variances were tested on model residuals. For normality assessment, probability–probability (P-P) plots were used, and for homoscedasticity, Levene’s test was used. Tukey’s HSD test was used for multiple comparisons following significant ANOVA results. Differences were considered statistically significant at *p* < 0.05 and highly significant at *p* < 0.01. All analyses were performed using Statistica 14.1.0.8 (TIBCO Software Inc., San Ramon, CA, USA).

## 3. Results

Total weight gain of larvae was significantly affected by feed protein level, while the effect of energy level was not significant (Table 4). Larvae fed higher protein levels gained weight faster. The difference was large between the low-protein and the other two groups, 16% on average, and was quite numerically consistent in all observed weeks, despite being statistically significant in some, and not in others. The difference between medium- and high-protein-level groups was numerically small and insignificant when observed week by week; yet, for the whole trial, this small difference (3%) was also found significant by post hoc test. A significant effect of energy on weight gain and a significant protein x energy interaction were observed in week 3, as a result of the HP-HE group gaining 12% slower than the other two HP groups. This difference was reduced to 6% in total and did not result in a statistically significant interaction for the whole period.

Feed intake was quite similar across most groups (Table 5). The only group with somewhat lower intake was the HP-LE (high protein-low energy) group. ANOVA revealed a significant effect of protein level on feed intake during the first two weeks, and a significant effect of energy level in the third week, last week, and over the total period. Although the HP-LE group exhibited reduced intake, the observed effect was very small (2%) and therefore of limited practical relevance. It is possible that the differences observed across energy levels are primarily driven by the reduced intake in this single group, potentially due to the higher inclusion of beet pulp in this feed (9%). Some statistically significant interactions of trial with protein and energy were also noted, yet they are hard to interpret and of little practical importance.

As a result of variable gain performance and quite stable feed intake, feed conversion ratio values (Table 6) are mainly reflecting weight gain differences (Table 4). Groups with higher gains are also having lower FCR, a pattern commonly observed in domestic animals. The protein effect on FCR was significant in four of the five weeks and in the whole period. Effects of energy and P × E interaction were present in week 3, again due to the HP-HE group performing much worse than the other two HP groups.

Besides the feed conversion ratio, the protein conversion ratio was also calculated. It was unaffected by energy level, like FCR, and significantly affected by protein level. It shows opposite variation compared with FCR. With increasing protein level, feed conversion ratio becomes better and protein conversion ratio becomes worse. This implies that, when faced with protein deficiency, larvae tend to use protein more efficiently. The protein conversion ratio difference was only significant between the HP and the other two groups.

The chemical composition of larvae, expressed on a dry matter basis, is shown in Table 7. The largest differences were observed in fat content, which ranged from 38.5% with the low-protein feed to just 26.9% with the high-protein feed. The effects of energy and the protein × energy interaction on fat content were not significant. In contrast to the fat content, variability in protein content was smaller, but both main effects and their interaction were significant. Larval protein content increased with the protein level in the feed, while the effect of energy was numerically small and non-linear.

The NDF content was significantly affected by energy level, with the low-energy group having higher NDF than the other two groups. In the case of ADF, both main effects were significant, although the numerical differences were small. ADF increased with increasing protein and decreased with increasing energy. Ash content was highly uniform, ranging from 3.53% in the LP-HE group to 3.93% in the HP-LE group; however, due to very low variation within groups, all effects were statistically significant. Increasing energy decreased ash content, while the protein effect was non-linear.

Feed price was mainly determined by its protein content, while increasing energy levels only mildly affected the cost (Table 8). This can be explained by the ingredients used—protein levels were increased using soybean meal, a costly component, while energy was adjusted using cheap high-energy feed-corn, and expensive low energy feed–sugar beet pulp.

From the standpoint of feed cost per unit of gain, medium levels performed best, especially in the case of protein. Low protein resulted in reduced gains, while high protein did not lead to sufficient improvement to justify the increased cost. Gain was only 3% higher, while the price was 7% higher for the high protein feed compared to the medium protein feed.

Feed cost per unit of protein gain was again predominantly affected by feed protein level, rather than energy. The difference between medium- and high-protein feeds was moderate, while the low-protein group performed consistently worse. It can be concluded that, despite better protein conversion when low-protein feed is used (Table 6), the cheapest protein production is achieved with higher protein levels (Table 8).

## 4. Discussion

Comparison of weight gain is quite challenging because experimental protocols for insects are not standardized. Researchers usually opt for one of the following approaches: relative growth, mean individual growth per number of individuals [35,36,37,38,39], or per container in which the insects are kept [29]. Our results are presented as the weight gain per tray in which larvae were kept. The results showed an effect of dietary protein level on the growth performance of the larvae (weight gain), whereas energy level showed no effect. Jajić et al. [26] found that more protein in the diet increased both larval protein content and body weight, and Montalbán et al. [37] also showed better growth and feed efficiency with higher-protein byproducts. López-Gámez et al. [40] even reported that larvae fed with certain protein-rich vegetable wastes almost doubled their weight compared to controls. On the other hand, Tamim et al. [41] noted that high energy levels mostly influenced fat storage rather than growth, which matches well with what was observed in our study. A recent review [42] also summarized that protein is the main driver of growth in *T. molitor*, while high energy levels just lead to more fat. Altogether, this supports the conclusion that protein level is the key factor for larval performance. The dietary energy level in our study was balanced by adding different amounts of dehydrated sugar beet pulp, which may have slightly increased the fiber content in some diets and affected digestibility. From our unpublished digestibility studies, we know that *Tenebrio molitor* larvae cannot really use sugar beet pulp as an energy source. Also, the amount of sugar beet pulp in the diets was relatively small, so its additive effect on overall nutrient levels was probably negligible. Therefore, it mainly acted as an energy diluent, although we cannot completely rule out that the extra fiber had some influence on larval performance.

The effect of dietary protein and energy levels on feed intake was minimal. As shown in Table 4, intake remained fairly consistent across all groups, with only the HP-LE group showing a slight reduction of about 2%. This small variation suggests that larvae did not respond to changes in diet composition by markedly adjusting how much they consumed, but rather through the differences in efficiency of nutrient use. Similar trends have been reported in previous research on *T. molitor*. Rho and Lee [25] found that larval growth was clearly affected by the level of protein in the diet, while energy level itself had little or no effect on feed intake. Their results point to the importance of protein as a limiting factor for growth, with larvae apparently not compensating for lower-protein diets by increasing intake. In line with this, Kröncke et al. [43] demonstrated that mealworms are capable of balancing their nutrient intake through selective feeding behavior, adjusting the quality rather than the quantity of food they consume. Taken together, these findings reinforce the view that in *T. molitor*, diet composition has a stronger influence on growth and nutritional outcomes than on total feed intake, which tends to remain relatively stable across dietary treatments.

Feed conversion ratio in *Tenebrio molitor* can vary quite a lot between studies, largely due to differences in substrate types, nutrient digestibility, and experimental methods. Reported FCRs for mealworms usually range from about 1.6 to 3.0 on cereal-based diets, but they can be much higher when larvae are fed low-quality or fiber-rich substrates [29]. This clearly illustrates the importance of diet composition for efficient nutrient use and biomass production. In our study, the lowest feed conversion ratio was achieved with the HP-ME diet, averaging 1.89 over the course of the experiment. The poorest FCR was observed with the LP-LE diet, with an average FCR of 2.34 throughout the trial period. These values fall within the typical feed conversion ratio range reported for *T. molitor* in most studies.

Controlled nutrient-balance experiments have shown that protein is especially important for regulating growth and feed conversion ratio. Rho and Lee [25] demonstrated that the protein-to-carbohydrate ratio strongly influences larval performance, with an optimum around 1.7–1.8:1 leading to faster growth and better efficiency. Our findings align with these results: protein levels had a noticeably stronger effect on larval growth than energy levels. Beyond controlled lab experiments, studies with practical feed substrates show similar trends. Kröncke et al. [43] found that larvae allowed to self-select among different feed ingredients grew faster and converted feed more efficiently, with reported FCRs ranging from approximately 1.6 to 3.0 depending on the diet composition.

Finally, reviews emphasize that optimizing the nutritional value of mealworms for animal feed depends not only on protein and energy levels, but also on substrate digestibility and rearing practices [42]. Taken together, these studies help explain why FCR values vary so widely across the literature. Our results fit well within this context, reinforcing that protein supply is a key factor in achieving efficient growth and feed conversion ratio in *T. molitor*.

The current focus on rearing insects centers around their potential as a protein source. However, insects’ bodies also contain other chemical compounds that can be influenced by their diet. The fat extracted from insects can serve as an energy source, but it generally contains polyunsaturated fatty acids that are prone to oxidation and spoilage [43,44]. Our objective was to achieve the highest possible percentage of protein while minimizing fat content. Insect lipids represent a component that is prone to rapid deterioration [45]. The use of proper optimization of larval diets, the lipid content can be reduced and directed toward a more desirable chemical composition of insects as an alternative protein source in animal nutrition. There is no doubt that diet influences the chemical composition of *T. molitor*. In their study investigating the optimal levels of protein and energy levels in the diet of *T. molitor* larvae, Rho and Lee [25] showed that increased dietary protein intake, up to a certain threshold, improved larval performance while reducing fat storage, while higher energy levels promoted fat accumulation but compromised growth and developmental performance. When larvae were fed six different diets with protein content from 11.4% to 22.6% (dry weight), the percentage of protein in the larvae ranged from 38.9% to 71.2%. This research indicates that diet significantly affects the chemical composition of *Tenebrio molitor* [46]. In another study, a statistically significant difference in the protein content of *T. molitor* was also shown, which ranged from 45.96% to 52.46%, due to dietary variations in feed mixtures containing 18.51% to 26.3% protein [38]. In line with these findings, Kronke et al. [43] reported that dietary protein content had a significant impact not only on growth performance but also on the nutritional composition of *T. molitor* larvae, indicating that dietary protein levels are a key factor in both production efficiency and the nutritional profile of larvae. Furthermore, from a practical perspective, López-Gámez et al. [40] pointed out that supplementing the diet of mealworms with plant-based byproducts can improve both growth performance and nutritional quality, supporting the potential of sustainable feed ingredients to optimize larval development while valorizing agro-industrial residues. The amount of ADF in insects is influenced by both energy and protein levels. Specifically, an increase in protein results in a higher ADF fraction, while a higher energy levels leads to a decrease in ADF. The ADF in insects is likely derived from chitin and is chemically similar to cellulose [47]. Chitin in insects may play a significant role in digestibility, as higher levels may decrease digestibility in animals such as poultry [48]. The production parameters in this study, such as weight gain and feed intake, are expressed per tray, which, although is less common than individual-based metrics, is an accepted approach in production-oriented studies aiming to evaluate overall system productivity under conditions relevant to large-scale insect rearing [29,40].

To obtain sustainable insect farming, this emerging industry will have to focus on economic parameters of production. This research shows that finding an economically optimal solution might be a challenging task involving integrating multiple important parameters. Contrary to the energy levels used in this research that all resulted in the similar feed costs and the similar production performances, results obtained with different protein levels were different and each offered an advantage of its own. Low-protein feed was cheapest and resulted in the best protein conversion rate. With the low-protein diets, just 1.81 g of feed protein was needed to produce 1 g of insect protein, compared to 1.97 g in high-protein diets. Contrary to that, protein production was cheaper using high-protein diets. That is because these diets contained 37% more protein for just 17% cost increase. That made them more economically valuable despite protein from them being used less efficiently. If focusing on the whole gain, not just proteins, medium protein level performed best in economical terms. Real-life decisions would have to be made considering current feed prices, insect performance results and both insect protein and insect fat prices.

## 5. Conclusions

The results of the current study show that the content of dietary protein is the primary regulator of growth, feed conversion, and nutritional content of *Tenebrio molitor* larvae and that the energy levels per se have an extremely limited impact. Both feed efficiency and weight gain were always higher for protein-rich diets, and high-energy-level diets promoted fat deposition without stimulating growth. Feed consumption was relatively stable across treatments, confirming that larvae control nutrient use rather than total consumption in response to diets of changing composition. Apart from growth performance, the level of dietary protein also had a profound effect on larval composition, i.e., protein content and ADF proportion, indicating its role in both nutritional quality and production efficiency. The findings confirm that protein is a limiting factor for mealworm performance, while high energy levels reduce efficiency and alter body composition.

From a practical perspective, optimizing protein supply from sustainable feed substrates not only enhances growth and conversion efficiency but also the nutritional value of larvae, justifying their utilization as a high-quality protein source in animal feeds. In general, this study reconfirms that specific dietary formulation, more especially protein level, is paramount for the optimization of *T. molitor* rearing system efficiency, nutritional value, and sustainability.

Protein supply is the primary nutritional driver of growth performance, feed efficiency, and body composition in *T. molitor* larvae. Increasing energy, without adequate protein levels, mainly promotes fat deposition rather than productive biomass gain. The economic effect of production should be considered during the formulation of feed mixtures for *T. molitor*, especially in the case of large-scale production.

## Figures and Tables

**Table 1:** The composition (%) of the raw material used in meals for *T. molitor*.

	Wheat Bran	Soybean Meal	Corn	Sugar Beet Pulp
Dry matter	90.88	88.94	87.09	89.33
Crude protein	16.73	44.45	8.92	8.98
Ether extract	4.1	2.15	3.58	0.41
CF	9.43	5.23	1.66	24.17
ASH	5.02	6.60	1.10	5.88
NFE	55.6	26.51	71.83	49.89

**Table 2:** Feed composition of experimental diets for *T. molitor* larvae (%).

	Wheat Bran	Soybean Meal	Corn	Sugar Beet Pulp
LP-LE	100	/	/	/
MP-LE	89	7	/	4
HP-LE	74	16	1	9
LP-ME	82	3	13	2
MP-ME	76	10	11	3
HP-ME	72	16	9	3
LP-HE	63	7	26	4
MP-HE	63	13	22	2
HP-HE	63	16	20	1

LP-LE: low protein-low energy; MP-LE: medium protein-low energy; HP-LE: high protein-low energy; LP-ME: low protein-medium energy; MP-ME: medium protein-medium energy; HP-ME: high protein-medium energy; LP-HE: low protein-high energy; MP-HE: medium protein-high energy; HP-HE: high protein-high energy.

**Table 3:** The composition of nine different meals for *T. molitor*.

	Crude Protein (%)	Ether Extract (%)	CF (%)	ASH (%)	NFE (%)	Digestible Energy (MJ/kg DM)
LP-LE	13.38	3.24	6.04	4.74	62.79	8.57
MP-LE	15.74	2.94	7.05	4.85	59.62	8.51
HP-LE	18.33	2.57	7.60	4.90	56.64	8.26
LP-ME	13.22	2.83	5.04	4.17	64.38	9.18
MP-ME	15.66	2.62	5.61	4.38	62.10	9.19
HP-ME	17.94	2.50	5.99	4.43	59.50	9.20
LP-HE	13.59	2.58	5.27	3.36	65.08	9.90
MP-HE	15.79	2.40	5.41	3.9	62.76	9.95
HP-HE	18.46	2.51	6.04	4.23	59.97	9.95

LP-LE: low protein-low energy; MP-LE: medium protein-low energy; HP-LE: high protein-low energy; LP-ME: low protein-medium energy; MP-ME: medium protein-medium energy; HP-ME: high protein-medium energy; LP-HE: low protein-high energy; MP-HE: medium protein-high energy; HP-HE: high protein-high energy, the estimation of the digestibility energy for corn, wheat bran and soybean meal taken from the Pascual et al. [29].

**Table 4:** Effect of protein and energy level on larval weight gain (g/day) per container.

	Week 1	Week 2	Week 3	Week 4	Week 5	Total
Protein						
Low	0.33 ^b^	0.35	0.64 ^b^	0.75 ^b^	0.86	0.58 ^c^
Medium	0.37 ^a^	0.39	0.71 ^a^	0.88 ^a^	0.97	0.66 ^b^
High	0.38 ^a^	0.40	0.74 ^a^	0.90 ^a^	1.02	0.68 ^a^
Energy						
Low	0.35	0.39	0.69	0.83	0.94	0.64
Medium	0.37	0.38	0.70	0.87	0.94	0.65
High	0.36	0.37	0.68	0.83	0.98	0.64
Protein × energy						
LP-LE	0.31	0.36	0.63 ^c^	0.71	0.86	0.57
MP-LE	0.34	0.39	0.68 ^bc^	0.87	0.99	0.65
HP-LE	0.39	0.42	0.76 ^a^	0.91	0.97	0.69
LP-ME	0.33	0.33	0.62 ^c^	0.78	0.86	0.58
MP-ME	0.39	0.39	0.72 ^ab^	0.90	0.92	0.67
HP-ME	0.39	0.42	0.78 ^a^	0.93	1.04	0.71
LP-HE	0.35	0.35	0.65 ^bc^	0.77	0.87	0.60
MP-HE	0.37	0.39	0.72 ^ab^	0.87	1.01	0.67
HP-HE	0.35	0.37	0.68 ^bc^	0.85	1.05	0.66
SEM	0.008	0.014	0.010	0.019	0.033	0.007
*p* values						
Protein	0.048 *	0.082	0.019 *	0.002 **	0.078	0.004 **
Energy	0.225	0.534	0.031 *	0.335	0.722	0.055
Trial	0.148	0.235	0.947	0.164	0.322	1.000
P × E	0.062	0.398	0.025 *	0.134	0.141	0.080
P × T	0.459	0.521	0.444	0.876	0.123	0.720
E × T	0.305	0.490	0.917	0.152	0.050	0.887
P × E × T	0.497	0.076	0.671	0.584	0.784	0.145

LP-LE: low protein-low energy; MP-LE: medium protein-low energy; HP-LE: high protein-low energy; LP-ME: low protein-medium energy; MP-ME: medium protein-medium energy; HP-ME: high protein-medium energy; LP-HE: low protein-high energy; MP-HE: medium protein-high energy; HP-HE: high protein-high energy. * indicates statistical significance at *p* < 0.05, while ** indicates statistical significance at *p* < 0.01. ^a–c^—Means within column subsections sharing the same letter do not differ significantly (*p* > 0.05). SEM—pooled standard error of the mean.

**Table 5:** Effect of protein and energy level on feed intake (g/day) per container.

	Week 1	Week 2	Week 3	Week 4	Week 5	Total
Protein						
Low	0.77 ^a^	0.94 ^a^	1.40	1.41	2.29	1.34
Medium	0.76 ^a^	0.93 ^a^	1.40	1.41	2.29	1.34
High	0.75 ^b^	0.91 ^b^	1.39	1.41	2.29	1.33
Energy						
Low	0.76	0.91	1.39 ^b^	1.40	2.28 ^b^	1.32 ^b^
Medium	0.77	0.92	1.40 ^a^	1.41	2.30 ^a^	1.34 ^a^
High	0.76	0.94	1.40 ^a^	1.42	2.30 ^a^	1.34 ^a^
Protein × energy						
LP-LE	0.76	0.94 ^ab^	1.40 ^a^	1.41	2.29 ^bc^	1.34
MP-LE	0.75	0.92 ^b^	1.39 ^a^	1.41	2.29 ^bc^	1.33
HP-LE	0.76	0.88 ^c^	1.37 ^b^	1.39	2.27 ^c^	1.31
LP-ME	0.77	0.93 ^ab^	1.40 ^a^	1.42	2.30 ^ab^	1.34
MP-ME	0.77	0.93 ^ab^	1.41 ^a^	1.41	2.30 ^ab^	1.34
HP-ME	0.76	0.92 ^ab^	1.40 ^a^	1.41	2.29 ^ab^	1.33
LP-HE	0.77	0.94 ^ab^	1.40 ^a^	1.41	2.30 ^ab^	1.34
MP-HE	0.76	0.95 ^a^	1.41 ^a^	1.41	2.30 ^ab^	1.34
HP-HE	0.73	0.93 ^ab^	1.40 ^a^	1.42	2.31 ^a^	1.34
SEM	0.003	0.006	0.005	0.003	0.005	0.003
*p* values						
Protein	0.012 *	0.029 *	0.108	0.523	0.307	0.053
Energy	0.199	0.378	0.047 *	0.078	0.034 *	0.005 **
Trial	0.950	0.841	0.755	1.000	0.118	1.000
P × E	0.814	0.018 *	0.043 *	0.121	0.003 **	0.143
P × T	0.965	0.480	0.347	0.825	0.162	0.557
E × T	0.638	0.014 *	0.388	0.539	0.052	0.925
P × E × T	0.000 **	0.718	0.650	0.024 *	0.983	0.200

LP-LE: low protein-low energy; MP-LE: medium protein-low energy; HP-LE: high protein-low energy; LP-ME: low protein-medium energy; MP-ME: medium protein-medium energy; HP-ME: high protein-medium energy; LP-HE: low protein-high energy; MP-HE: medium protein-high energy; HP-HE: high protein-high energy. * indicates statistical significance at *p* < 0.05, while ** indicates statistical significance at *p* < 0.01. ^a–c^—Means within column subsections sharing the same letter do not differ significantly (*p* > 0.05). SEM—pooled standard error of the mean.

**Table 6:** Effect of protein and energy level on feed and protein conversion ratios (g/g).

	Week 1	Week 2	Week 3	Week 4	Week 5	Total FCR	Total PCR
Protein							
Low	2.33 ^b^	2.74 ^b^	2.22 ^b^	1.88 ^b^	2.68	2.29 ^c^	1.82 ^a^
Medium	2.10 ^a^	2.40 ^a^	1.99 ^a^	1.61 ^a^	2.38	2.02 ^b^	1.86 ^a^
High	2.01 ^a^	2.28 ^a^	1.90 ^a^	1.58 ^a^	2.26	1.94 ^a^	1.97 ^b^
Energy							
Low	2.21	2.37	2.02	1.72	2.45	2.09	1.90
Medium	2.09	2.47	2.02	1.64	2.48	2.07	1.86
High	2.14	2.57	2.07	1.72	2.39	2.09	1.88
Protein × energy							
LP-LE	2.43	2.64	2.22 ^cd^	1.99	2.66	2.34	1.87
MP-LE	2.23	2.36	2.05 ^bc^	1.62	2.34	2.04	1.84
HP-LE	1.97	2.12	1.81 ^a^	1.54	2.34	1.91	1.99
LP-ME	2.33	2.84	2.27 ^d^	1.83	2.70	2.30	1.76
MP-ME	1.97	2.37	1.97 ^ab^	1.58	2.52	2.02	1.92
HP-ME	1.97	2.20	1.81 ^a^	1.53	2.23	1.89	1.90
LP-HE	2.23	2.72	2.17 ^bc^	1.84	2.68	2.24	1.83
MP-HE	2.09	2.47	1.96 ^ab^	1.63	2.29	2.00	1.81
HP-HE	2.09	2.53	2.09 ^bc^	1.68	2.21	2.04	2.01
SEM	0.061	0.086	0.048	0.041	0.084	0.021	0.029
*p* values							
Protein	0.039 *	0.038 *	0.020 *	0.001 **	0.080	0.005 **	0.004 **
Energy	0.525	0.435	0.016 *	0.386	0.785	0.063	0.698
Trial	0.293	0.503	1.000	0.288	0.125	1.000	-
P × E	0.117	0.478	0.035 *	0.117	0.267	0.060	0.183
P × T	0.375	0.668	0.453	0.970	0.133	0.553	-
E × T	0.091	0.312	0.974	0.231	0.052	0.948	-
P × E × T	0.585	0.108	0.558	0.448	0.744	0.162	-

LP-LE: low protein-low energy; MP-LE: medium protein-low energy; HP-LE: high protein-low energy; LP-ME: low protein-medium energy; MP-ME: medium protein-medium energy; HP-ME: high protein-medium energy; LP-HE: low protein-high energy; MP-HE: medium protein-high energy; HP-HE: high protein-high energy. * indicates statistical significance at *p* < 0.05, while ** indicates statistical significance at *p* < 0.01. ^a–d^—Means within column subsections sharing the same letter do not differ significantly (*p* > 0.05). SEM—pooled standard error of the mean.

**Table 7:** Effect of protein and energy level on larvae chemical composition (% dry matter).

	Crude Protein	Fat	NDF	ADF	Ash
Protein	45.6 ^bc^	38.5 ^a^	9.0	5.70 ^b^	3.68 ^ab^
Low	47.0 ^b^	33.6 ^b^	9.1	6.07 ^ab^	3.65 ^b^
Medium	50.6 ^a^	26.9 ^c^	9.3	6.14 ^a^	3.73 ^a^
High					
Energy	48.8 ^a^	33.1	9.8 ^a^	6.29 ^a^	3.82 ^a^
Low	46.4 ^b^	33.5	9.0 ^b^	5.89 ^ab^	3.69 ^b^
Medium	48.0 ^a^	32.5	8.7 ^b^	5.73 ^b^	3.55 ^c^
High	45.6 ^c^	38.5 ^a^	9.0	5.70 ^b^	3.68 ^ab^
Protein × energy					
LP-LE	46.3 ^bc^	40.4	9.7	6.00	3.79 ^b^
MP-LE	48.3 ^b^	32.5	9.7	6.38	3.73 ^bc^
HP-LE	51.8 ^a^	26.3	10.1	6.51	3.93 ^a^
LP-ME	45.5 ^bc^	37.5	9.1	5.60	3.71 ^bcd^
MP-ME	45.5 ^bc^	33.7	8.8	5.93	3.65 ^cde^
HP-ME	48.0 ^bc^	29.4	9.1	6.15	3.72 ^bc^
LP-HE	44.8 ^c^	37.6	8.4	5.51	3.53 ^e^
MP-HE	47.2 ^bc^	34.7	8.8	5.91	3.58 ^de^
HP-HE	51.9 ^a^	25.2	8.8	5.78	3.54 ^e^
SEM	0.69	1.21	0.29	0.21	0.03
*p* values					
Protein	0.000 **	0.000 **	0.418	0.036 *	0.009 **
Energy	0.001 **	0.559	0.000 **	0.010 *	0.000 **
P × E	0.040 *	0.057	0.885	0.934	0.004 **

LP-LE: low protein-low energy; MP-LE: medium protein-low energy; HP-LE: high protein-low energy; LP-ME: low protein-medium energy; MP-ME: medium protein-medium energy; HP-ME: high protein-medium energy; LP-HE: low protein-high energy; MP-HE: medium protein-high energy; HP-HE: high protein-high energy. * indicates statistical significance at *p* < 0.05, while ** indicates statistical significance at *p* < 0.01. ^a–e^—Means within column subsections sharing the same letter do not differ significantly (*p* > 0.05). SEM—pooled standard error of the mean.

**Table 8:** Effect of protein and energy on the feed cost of larvae and larval protein production.

	Feed Cost (EUR/kg)	Feed Cost per Gain (EUR/kg)	Feed Cost per Protein Gain (EUR/kg)
Protein			
Low	0.23	0.52	3.11
Medium	0.25	0.50	2.91
High	0.27	0.52	2.87
Energy			
Low	0.25	0.52	2.98
Medium	0.25	0.51	2.97
High	0.25	0.53	2.97
Protein × energy			
LP-LE	0.22	0.52	3.10
MP-LE	0.24	0.50	2.81
HP-LE	0.27	0.52	2.98
LP-ME	0.23	0.52	3.07
MP-ME	0.25	0.50	3.01
HP-ME	0.27	0.50	2.80
LP-HE	0.24	0.53	3.14
MP-HE	0.25	0.51	2.90
HP-HE	0.26	0.54	2.85

LP-LE: low protein-low energy; MP-LE: medium protein-low energy; HP-LE: high protein-low energy; LP-ME: low protein-medium energy; MP-ME: medium protein-medium energy; HP-ME: high protein-medium energy; LP-HE: low protein-high energy; MP-HE: medium protein-high energy; HP-HE: high protein-high energy.

## Data Availability

The data that support the findings of this study are available from the corresponding author upon reasonable request.
